# Tranilast Reduces Intestinal Ischemia Reperfusion Injury in Rats Through the Upregulation of Heme-Oxygenase (HO)-1

**DOI:** 10.3390/jcm14093254

**Published:** 2025-05-07

**Authors:** Emilio Canovai, Ricard Farré, Gert De Hertogh, Antoine Dubois, Tim Vanuytsel, Jacques Pirenne, Laurens J. Ceulemans

**Affiliations:** 1 Leuven Intestinal Failure and Transplantation Center (LIFT), University Hospitals Leuven, 3000 Leuven, Belgium; emilio.canovai@ouh.nhs.uk (E.C.); gert.dehertogh@uzleuven.be (G.D.H.); antoine.dubois@kuleuven.be (A.D.); tim.vanuytsel@uzleuven.be (T.V.); jacques.pirenne@uzleuven.be (J.P.); 2 Department of Abdominal Transplant Surgery, University Hospitals Leuven, 3000 Leuven, Belgium; 3 Department of Microbiology, Immunology and Transplantation, KU Leuven, 3000 Leuven, Belgium; 4 Translation Research Center for Gastrointestinal Disorders (TARGID), Department of Chronic Diseases and Metabolism (CHROMETA), KU Leuven, 3000 Leuven, Belgium; ricard.farre@kuleuven.be; 5 Translational Cell and Tissue Research, Department of Imaging & Pathology, KU Leuven, 3000 Leuven, Belgium; 6 Department of Gastroenterology and Hepatology, University Hospitals Leuven, 3000 Leuven, Belgium; 7 Department of Thoracic Surgery, University Hospitals Leuven, 3000 Leuven, Belgium; 8 Department of Chronic Diseases and Metabolism (CHROMETA), Laboratory of Respiratory Diseases and Thoracic Surgery (BREATHE), KU Leuven, 3000 Leuven, Belgium

**Keywords:** intestinal ischemia reperfusion injury, Tranilast, heme oxygenase-1

## Abstract

**Background:** Intestinal ischemia reperfusion injury (IRI) is a harmful process that occurs during intestinal infarction and intestinal transplantation (ITx). It is characterized by severe inflammation which disrupts the mucosal barrier, causing bacterial translocation and sepsis. Tranilast (N-[3,4-dimethoxycinnamoyl]-anthranilic acid) (TL) is a synthetic compound with powerful anti-inflammatory properties. **Objective:** To investigate the effect of pretreatment with TL in a validated rat model of intestinal IRI (60 min of ischemia). **Methods:** TL (650 mg/kg) was administered by oral gavage 24 and 2 h before the onset of ischemia. Experiment 1 examined 7-day survival in 3 study groups (sham, vehicle+IRI and TL+IRI, n = 10/group). In Experiment 2, the effects on the intestinal wall integrity and inflammation were studied after 60 min of reperfusion using 3 groups (sham, IRI and TL+IRI, n = 6/group). The following end-points were studied: L-lactate, intestinal fatty acid-binding protein (I-FABP), histology, intestinal permeability, endotoxin translocation, pro- and anti-inflammatory cytokines and heme oxygenase-1 (HO-1) levels. Experiment 3 examined the role of HO-1 upregulation in TL pretreatment, by blocking its expression using Zinc protoporphyrin (ZnPP) at 20 mg/kg vs. placebo (n = 6/group). **Results:** Intestinal IRI resulted in severe damage of the intestinal wall and a 10% 7-day survival. These alterations led to endotoxin translocation and upregulation of pro-inflammatory cytokines. TL pretreatment improved survival up to 50%, significantly reduced inflammation and protected the intestinal barrier. The HO-1 inhibitor ZnPP, abolished the protective effect of TL. **Conclusions:** TL pretreatment improves survival by protecting the intestinal barrier function, decreasing inflammation and endotoxin translocation, through upregulation of HO-1.This rat study of severe intestinal ischemia reperfusion injury demonstrates a novel role for Tranilast as a potential therapy. Administration of Tranilast led to a marked reduction in mortality, inflammation and intestinal permeability and damage. The study proved that Tranilast functions through upregulation of heme oxygenase-1.

## 1. Introduction

The intestinal tract is a highly complex structure that permits safe absorption of nutrients necessary to sustain life [1]. A single layer of tightly linked epithelial cells form a barrier between the intestinal lumen and underlying innate immune cells [2]. Damage to its integrity, like intestinal ischemia, can lead to bacterial translocation, sepsis and multiple organ failure [3]. Intestinal ischemia is caused by both acute arterial occlusions [4,5] such as embolism or atherosclerotic plaque ruptures-and ‘low flow’ ischemia due to sepsis, major vascular surgery or cardiopulmonary bypass [6,7]. Due to its insidious initial presentation and lack of specific treatment, mortality rates of up to 80% have been reported [8,9]. International guidelines recommend rapid revascularization and resection of all necrotic bowel [10,11]. However, restoring circulation to the bowel is not harmless, and will lead to additional injury through a process known as intestinal ischemia reperfusion injury (IRI) [8,12]. During ischemia, reactive oxygen species (ROS) are generated, leading to tissue damage driven by oxidative stress. When oxidative stress reaches critical levels, it causes lipid and protein oxidation in cell membranes, resulting in cellular dysfunction [13]. Oxidative reactions also induce DNA damage, contributing to long-term injury [14]. Reperfusion, the restoration of oxygen supply, can exacerbate this damage. Similar pathophysiological mechanisms are observed in other organs subjected to acute ischemia and sudden revascularization [15,16].

This damage disrupts the mucosal barrier, leading to bacterial translocation and sepsis [3,8,12]. Currently, treatment is mostly focused on rapid revascularization and supportive therapy (IV fluid resuscitation and broad-spectrum antibiotics) rather than directly treating the underlying IRI pathophysiology.

In Intestinal transplantation (ITx), a life-saving procedure for patients with complicated irreversible intestinal failure [17], there is an inevitable period of cold and warm ischemia followed by reperfusion that causes damage and can potentially compromise the graft function [18]. Specifically, the production of ‘danger signals’ caused by IRI prime the innate and adaptive immune system of the graft, provoking rejection [19]. Reducing this damage is critical to improve both short- and long-term survival.

Tranilast (N-[3,4-dimethoxycinnamoyl]-anthranilic acid)(TL) is a commercially available drug with strong anti-inflammatory properties [20]. It induces heme oxygenase-1 (HO-1) upregulation, a well-conserved anti-inflammatory pathway in all cells [21]. It blocks production of pro-inflammatory cytokines (interleukin (IL)-1β, IL-6 and tumor necrosis factor-*α* *(TNF-α)* by downregulating the *NF*-*κB* pathway [22]. Secondly, it is also a powerful anti-oxidant [23], which can help to reduce ROS that are formed during intestinal IRI [13,24]. Furthermore, it has been shown to have immunomodulatory effects, by inducing regulatory T cells (T-regs) [25]. TL pretreatment has already been shown to reduce IRI in different organs such as kidneys and brains [26,27]. However, the effect of TL pretreatment on intestinal IRI has not been evaluated.

We hypothesized that oral TL pretreatment could attenuate the damage caused by intestinal IRI in a pre-clinical rat model of intestinal IRI, partly through the up-regulation of HO-1.

## 2. Methods

### 2.1. Animal Model

We used the validated rat model of intestinal IRI previously described by our group [28]. Briefly, intestinal IRI was induced in male Sprague Dawley rats (n = 54, weight 275–325 g) (Janvier Labs, Saint Berthevin Cedex, Le Genest-Saint-Isle, France) by clamping the superior mesenteric artery for 60 min. Clamping was achieved through a midline laparotomy after general anesthesia delivered through intraperitoneal injection with a mixture of xylazine (10 mg/kg of Xyl-M2%, Van Miert & Dams Chemie, Arendonk, Belgium) and ketamine (100 mg/kg, Anesketin, Eurovet, Bladel, The Netherlands). The abdomen was temporarily closed and after 60 min the clamp was removed leading to reperfusion. This interval was chosen based on previous studies by our group [28,29].

The sham group underwent the same procedure as the other groups (including anesthesia and surgery) with the exception of clamping the superior mesenteric artery. At the end of the experiments, animals were sacrificed through exsanguination under general anesthesia. All animals underwent necropsy at time of death or sacrifice. Animals were housed in a dedicated facility following European Union guidelines on animal welfare, which included daily monitoring and postoperative analgesia using buprenorphine (Vetergesic, Ceva Animal Health Inc., Guelph, ON, Canada). Animals were allowed full access to water and food prior and after surgery. A standardized morbidity score was utilized registering signs of distress and resulting in euthanasia if too high [28]. None of the animals had to be euthanized in this study. Treated animals received either Tranilast (Sigma-Aldrich, Overijse, Belgium) at 650 mg/kg (dissolved in 1.4% NaHCO_3_ solution) or equivalent volume of vehicle only (1.4% NaHCO_3_ solution). Administration was performed through oral gavage at 24 and 2 h prior to start of ischemia.

The experiments were approved by the University of Leuven Ethics Committee for Animal Experimentation (EC P120/2016—approval date 1 September 2015).

### 2.2. Experimental Design

**Figure 1:**
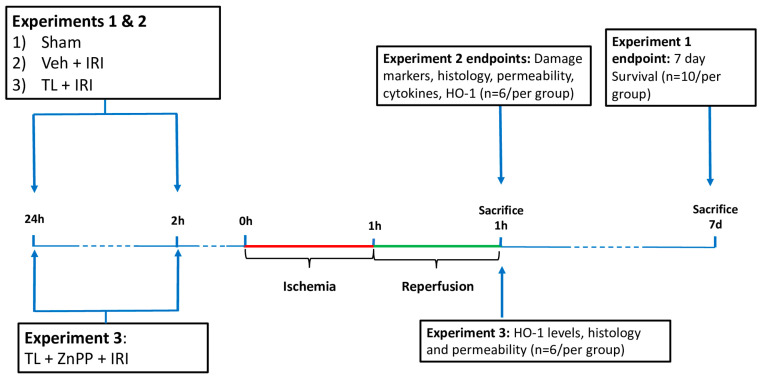
Overview of the study. IRI: ischemia reperfusion injury; TL: tranilast; HO-1: heme oxygenase-1; Veh: vehicle; ZnPP: zinc protoporphyrin.

This study involved three separate experimental set-ups (Figure 1). All experiments were performed by a single not blinded operator. The primary outcome was 7-day survival (Experiment 1). Secondary outcomes included histological damage, intestinal permeability, inflammatory marker levels, and the impact of HO-1 inhibition on the protective effects of TL.

**Experiment 1:** The effect of TL pretreatment (administration before induction of ischemia) on survival was studied. Rats were randomly divided into three groups (n = 10 per group): 1/sham, 2/Vehicle + IRI and 3/TL + IRI. After IRI, rats were observed for 7 days. In these animals, the abdomen was closed in two layers using non-resorbable sutures. The 7-day survivors were sacrificed for analysis. If an animal died during these 7 days, the cause was ascertained through necropsy.

**Experiment 2:** In these experiments, the effect of TL pretreatment on various endpoints related to intestinal wall structure and inflammation was assessed. Three groups were studied (n = 6 per group, randomly divided): 1/sham: anesthesia and surgery, without ischemia; 2/Vehicle + IRI: oral administration of vehicle (1.4% NaHCO_3_) 24 and 2 h prior to start of intestinal ischemia, 60 min ischemia and 60 min reperfusion; and 3/TL + IRI: oral administration of TL (650 mg/kg) 24 and 2 h prior to start of intestinal ischemia, 60 min ischemia and 60 min reperfusion. Dosing and timing of administration of TL were based on previously published data [20,30]. Animals were sacrificed by exsanguination under general anesthesia after 60 min of reperfusion. The studied end-points were plasma biomarkers for enterocyte damage (L-lactate, Intestinal fatty acid-binding protein (I-FABP)), histology (Park-Chiu score), epithelial barrier function (Ussing chambers technique), endotoxin translocation (Limulus Amebocyte Lysate Pyrogent kit, Lonza Bioscience, Basel, Switzerland), expression of tissue pro- and anti-inflammatory cytokines (quantitative reverse-transcription polymerase chain reaction (qRT-PCR)), and expression of HO-1 levels (Western blot (WB)).

**Experiment 3:** Finally, the role of TL induced HO-1 upregulation was studied. In an additional group of rats, Zinc protoporphyrin (ZnPP) (Sigma-Aldrich, Belgium)-a powerful inhibitor of HO-1 upregulation was administered in addition to TL. ZnPP was administered via intraperitoneal injection at 20 mg/kg, 24 h prior to IRI. This dosing and timing were based on previous studies [31].

The sample sizes for each group was based on a power calculation extrapolated from the expected protective outcomes seen in our previous study using the same IRI model [28]. Briefly, for experiment 1 (survival), we predicted an improvement in survival of around 50% (compared to 0%). For experiment 2 and 3, we used our histological damage score (Park-Chiu—range 0–8) and predicted a reduction by 2.5 points (See Supplemental File S1).

### 2.3. Sample Collection

After sacrifice, arterial blood was spun at 3500 rpm for 10 min and the isolated serum was stored at −80 °C after snap freezing in liquid nitrogen. Following a standardized protocol, 5 segments of distal ileum were collected (one segment of 5 cm and 4 segments of 1 cm long, starting from the ileo-cecal valve). The long segment was stored in 10 mM Glucose in Krebs-Ringer bicarbonate buffer and mounted in an Ussing chamber to measure permeability [32,33]. Three segments were snap frozen for qRT-PCR and WB analysis. Finally, one segment was fixed in 4% neutral-buffered formalin for histological analysis.

### 2.4. Damage Biomarkers

A sample (100 µL) of arterial blood was analyzed for L-Lactate using a blood gas analyzer (ABL-815, Radiometer, Copenhagen, Denmark). I-FABP, a marker of enterocyte damage, was measured by WB in the serum (see below) [34].

### 2.5. Histology

Ileal tissue samples were cut in 1 longitudinal and 2 transverse sections after fixation, properly oriented and embedded in paraffin. The tissue blocks were then cut into 5 µm-thick sections which were mounted on glass slides, stained with hematoxylin and eosin following the standard lab protocol, and coverslipped. The samples were analyzed, in a blinded fashion, by an experienced pathologist (GDH). The standardized analysis involved inspecting 4 separate fields as previously described [28]. Each field was independently graded for IRI related damage by measuring the average villus length (from tip of the villi to the mouths of the crypts, measured in µm) and the validated Park-Chiu IRI scoring system (0–8) [28,35].

### 2.6. Epithelial Barrier Assessment

In order to evaluate the effect of IRI on intestinal wall integrity, three ileal segments were mounted in an Ussing chamber setup (Mussler Scientific Instruments, Aachen, Germany). The aim was to measure the trans-epithelial electrical resistance (TEER) as an objective parameter of epithelial intestinal permeability to ions [32,33]. Briefly, the bowel segment was opened longitudinally and mounted in the setup with exposure to 10 mM Mannitol and 10 mM Glucose in Krebs-Ringer bicarbonate buffers on the mucosal and serosal sides respectively.

The tissue was kept oxygenated and at 37 °C. After 30 min stabilization, the average TEER was measured over a period of 90 min. TEER was corrected for villus length to compensate for areas of patchy necrosis. This was done by multiplying the original TEER by the ratio of the corresponding villus length by the mean sham villus length [28].

### 2.7. Bacterial Translocation

The plasma level of lipopolysaccharide (LPS) endotoxin levels was measured using a colorimetric Limulus Amebocyte Lysate (LAL QCL1000) Enzyme-Linked Immunosorbent Assay (ELISA) (Lonza, Bazel, Switzerland). Plasma LPS is a reflection of the transcellular epithelial passage of large molecules from the intestinal lumen to the blood [36].

### 2.8. Quantitative Reverse-Transcription Polymerase Chain Reaction (qRT-PCR)

In these analyses, the relative expression of both pro-inflammatory cytokines (Interleukin (IL)-1β, IL-6, interferon (IFN)-γ, tumor necrosis factor (TNF)-α) and anti-inflammatory cytokines (IL-10 and IL-13) was determined. Stored ileal tissue was homogenized in TRIzol reagent (Life Technologies, Carlsbad, CA, USA) and total RNA was isolated using RNeasy Minikit (Qiagen, Antwerp, Belgium) per instructions of the manufacturer. Next, c-DNA was created from 200 ng RNA using M-MLV transcriptase (Life-Technologies, Carlsbad, CA, USA). Finally, the relative expression of pro- and anti-inflammatory cytokines were determined by qRT-PCR using a LightCycler 96 W (Roche, Vilvoorde, Belgium) with Taqman Fast Universal PCR Master Mix and Taqman Gene Expression Assays (Life-Technologies, CA, USA) (IL-6 (Rn01410330_m1), IL-1β (Rn00580432_m1), TNF-α, (Rn00562055) IFN-γ (Rn00594078), IL-10 (Rn00563409) and IL-13 (Rn00587615)). Amplification was performed in three steps: 95 °C for 10 min followed by 45 cycles of amplification (95 °C for 10 s, 60 °C for 15 s, 72 °C for 10 s) and finally a melting curve program. Target messenger RNA (mRNA) expression was quantified relative to the house- keeping gene GAPDH (Rn01775763_g1; Life Technologies, CA, USA).

Cytokine data are shown as fold changes compared to the sham data of their respective series (i.e., mean sham value is 1).

#### Western Blot

Ileal (HO-1) and plasma samples (I-FABP) were used for these analyses. Ileal samples were first homogenized using RIPA buffer (50 mM Tris/HCl, 150 mM sodium chloride, 1% IPEGAL, 1 mM ethylenediamine tetra-acetic acid, 1% Protease Inhibitor Cocktail (Sigma- Aldrich, Overijse, Belgium), 1% Phosphatase Inhibitor Cocktail 2 and 3 (Sigma-Aldrich, St. Louis, MO, USA).

Protein concentration was measured by a standard Bradford assay (Sigma-Aldrich, MO, USA). Samples (50 μg protein) were subjected to sodium dodecyl sulfate polyacrylamide gel electrophoresis (SDS-PAGE) using Any kDa Mini-Protean TGX Precast Gels (Bio-Rad, Lokeren, Belgium) and proteins were blotted on polyvinylidene difluoride (PVDF) membranes using the Transblot Turbo system (Bio-Rad, Hercules, CA, USA).

For the plasma samples, the membranes were incubated with 0.1% Ponceau S staining solution. The membranes were then blocked for 1 h at room temperature with PBS-Tween (0.1%) containing 5% milk powder and incubated with the primary antibody (FABP2 (21252-1-AP, Proteintech Europe, Manchester, UK)); HO-1; GAPDH (G8795, Sigma-Aldrich, MO, USA) antibody overnight at 4 °C. After a 1-h incubation with the secondary antibody (anti-rabbit IgG HRP-linked antibody for FABP2 and HO-1 and anti-mouse IgG HRP-linked antibody (all Cell Signaling Technologies, Danvers, MA, USA), the proteins were detected using enhanced chemiluminescence (Pierce ECL Western Blotting Substrate) and digital detection with the Chemidoc MP system. Quantification of relative band intensity was then performed with the associated ImageLab software (Bio-Rad, CA, USA).

### 2.9. Statistical Analysis

The data in the text is shown as mean + standard deviation. All data were tested for normality using the Kolmogorov-Smirnov test. Multiple group comparisons were performed using One-Way ANOVA and post-hoc Tukey test in case of normal distribution or Kruskal-Wallis with post-hoc Dunn test for non-normal distribution. Kaplan-Meier estimator was used for survival analysis (log-rank test). A *p* value < 0.05 was considered statistically significant. All data sets were screened for outliers using the Robust regression and OUTlier removal (ROUT) technique using a coefficient of 5% [37]. In graphs, the middle line represents the mean and the whiskers indicate the standard error of the mean. GraphPad Prism version 9.0 (San Diego, CA, USA) was used for all analyses and graphs.

## 3. Results

### 3.1. Experiment 1: Effect of Tranilast Pretreatment on Survival

#### TL Pretreatment Improved Seven-Day Survival After IRI

**Figure 2:**
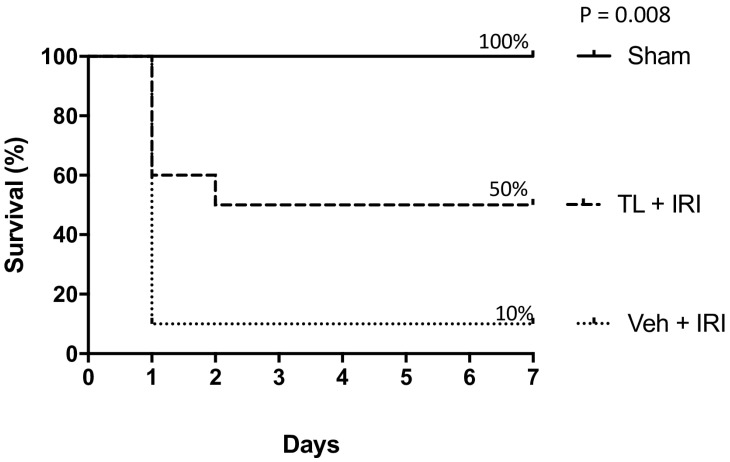
Tranilast improves 7-day survival after intestinal IRI. TL: tranilast; IRI: ischemia reperfusion injury. n = 10/group; log-rank: *p* = 0.008.

All sham rats survived the surgery and promptly recovered. IRI led to severe sepsis and early death (<24 h) in 9 out of 10 rats (Figure 2). All these animals succumbed to multi-organ failure and sepsis. In contrast, 5 rats pre-treated with TL survived for 7 days (*p* = 0.008). Four died within 24 h (severe sepsis) and one died at day 2 (intestinal perforation). Necropsy revealed a profoundly necrotic bowel with several perforations in all rats that died before 7 days. This extreme damage of the bowel wall led to endotoxin translocation and death. At sacrifice after 7 days, all surviving rats from the sham group had a normal aspect of the bowel. The surviving rat from the IRI-group presented with a generally normal aspect of the bowel but with severe adhesions. The starting weight was 312 g (±17) and the end-weight of survivors was 341 (±29). Weight evolution did not correlate with survival.

### 3.2. Experiment 2: Effect of Tranilast Pretreatment on Morphology, Barrier Function and Immune Activation

#### 3.2.1. TL Pretreatment Reduced IRI Induced Epithelial Damage

**Figure 3:**
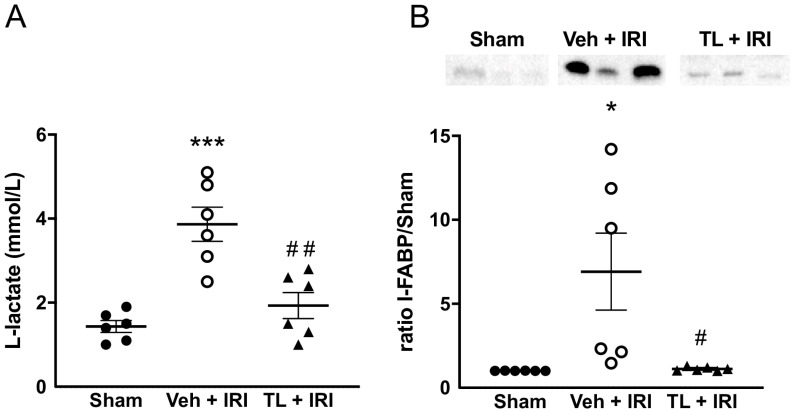
Tranilast reduces plasmatic release of intestinal IRI markers. (**A**) L-lactate; (**B**) I-FABP. I-FABP: intestinal-fatty acid binding protein; IRI: ischemia reperfusion injury; TL: tranilast; Veh: vehicle. *: *p* < 0.05 (compared to Sham); ***: *p* < 0.001 (compared to Sham), #: *p* < 0.05 (compared to Vehicle); ##: *p* < 0.01 (compared to Vehicle).

L-lactate levels were increased after IRI compared to the sham group (3.87 mmol/L ±1.00 vs. 1.43 ± 0.34 mmol/L, *p* < 0.0005) (Figure 3A). Pretreatment with TL reduced circulating L-lactate levels to near-to-normal values (1.93 ± 0.76 mmol/L, *p* = 0.012).

I-FABP showed a similar pattern as L-lactate. Its expression was very low in the sham rats, but it was higher after IRI (6.91 ± 5.62 fold increased, *p* = 0.0171). TL pretreatment led to a marked reduction to levels equal to the sham group (1.13 ± 0.11, *p* = 0.0192 compared IRI group) (Figure 3B).

#### 3.2.2. TL Pretreatment Reduced IRI Induced Intestinal Wall Damage

**Figure 4:**
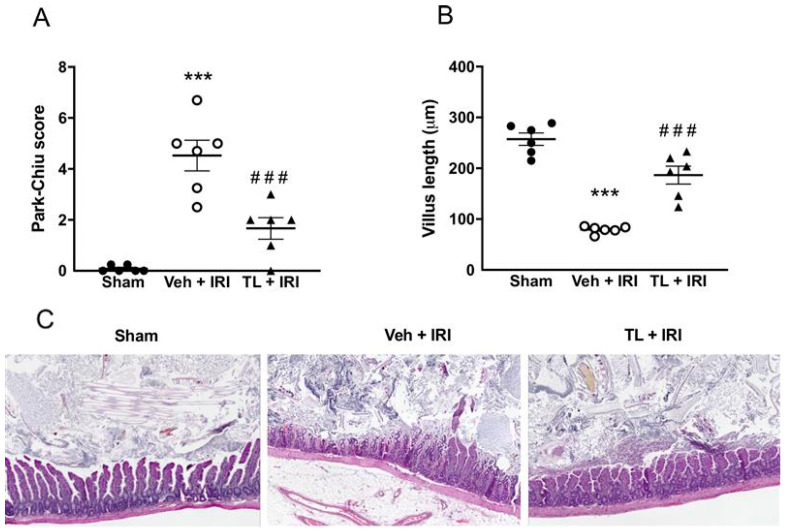
Tranilast protects the intestinal mucosal wall integrity as shown by (**A**) Park-Chiu score and (**B**) villus-length. (**C**) Representative histological illustration for each group, Hematoxylin and Eosin staining, magnification: X80. IRI: ischemia reperfusion injury; TL: tranilast; Veh: vehicle; ***: *p* < 0.001 (compared to sham), ^###^: *p* < 0.001 (compared to Vehicle).

Sham rats had normal intestinal histology, with a Park-Chiu score of 0 and a villus length of 257 μm ± 30 (Figure 4A,B). Intestinal IRI led to pronounced damage to the intestinal wall, resulting in a Park-Chiu score of 4.53 ± 1.48 which was reduced in the TL + IRI group (1.67 ± 1.03, *p* = 0.0007). Similarly, IRI induced damage which resulted in a marked reduction in villus length (79 μm ± 7, *p* < 0.0005 compared to sham). Villus length was preserved in TL pretreated rats (187 μm ± 43, *p* < 0.0005 compared to vehicle). On classic Hematoxylin and Eosin staining, IRI resulted in loss of villus integrity and severe interstitial edema (Figure 4C). This was attenuated in the TL TL + IRI group.

#### 3.2.3. TL Pretreatment Reduced Epithelial Permeability of the Intestine

**Figure 5:**
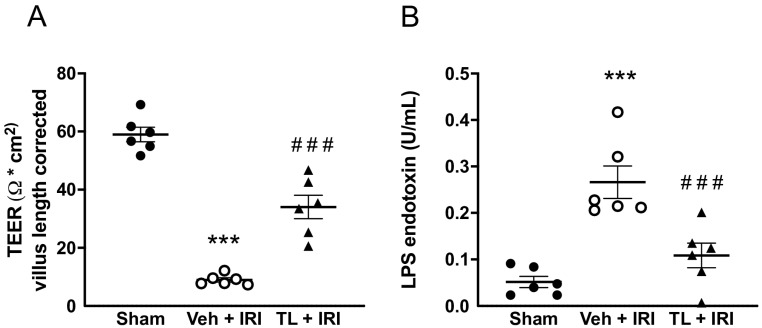
Tranilast ameliorates the intestinal barrier function as shown by: (**A**) increased TEER as measured in the Ussing Chamber; (**B**) reduced LPS endotoxin translocation into the blood. IRI: ischemia reperfusion injury; LPS: lipopolysaccharide, TEER: transepithelial electrical resistance, TL: tranilast; Veh: vehicle; ***: *p* < 0.001 (compared to Sham). ^###^: *p* < 0.001 (compared to Vehicle).

Mirroring the morphological results, IRI also increased the epithelial paracellular permeability as shown by a reduced TEER. TEER was lower in the vehicle group compared to sham (9 ± 2 vs. 59 ± 6 Ω.cm^2^, *p* < 0.0005) (Figure 5A). TL pretreatment led to an increase in TEER (34 Ω.cm^2^ ± 10, *p* < 0.0005 vs. vehicle). Moreover, we assessed the transcellular permeability by measuring circulating endotoxin. Translocation of endotoxin increased after IRI (0.27 U/L ± 0.09 vs. 0.05 U/L ± 0.03 in the sham group, *p* < 0.0005). TL pretreatment led to lower endotoxin levels (0.11 U/L ± 0.06, *p* = 0.0019) compared to vehicle (Figure 5B).

#### 3.2.4. TL Pretreatment Reduced Pro-Inflammatory and Increased Anti-Inflammatory Cytokines

**Figure 6:**
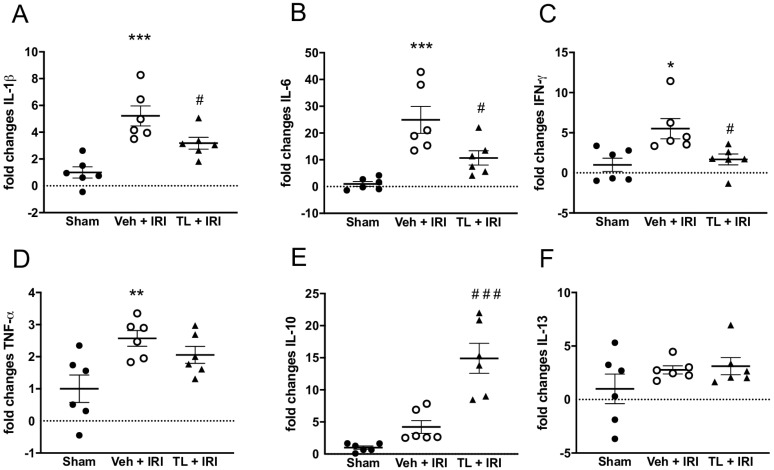
The anti-inflammatory effect of Tranilast in intestinal IRI as shown by (**A**) mRNA expression of pro-inflammatory cytokines IL-1β; (**B**) IL-6; (**C**) IFN-γ; and (**D**) TNF-α. mRNA expression of anti-inflammatory cytokines (**E**) IL-10 increased with Tranilast while (**F**) IL-13 did not differ between the different groups. IFN-γ: interferon-gamma; IL: interleukin; IRI: ischemia reperfusion injury; TL: tranilast; TNF-α: tumor necrosis factor- α; Veh: vehicle; *: *p* < 0.05 (compared to Sham); **: *p* < 0.01; ***: *p* < 0.001, ^#^: *p* < 0.05 (compared to Vehicle); ^###^: *p* < 0.001.

Intestinal IRI led to increased inflammation as evidenced by an upregulation of (i) IL-1β in ileal samples (5.22 ± 1.82 vs. 3.18 ± 1.07 fold; *p* < 0.0005), and (ii) plasmatic IL-6 (24.94 ± 12.37 vs. 10.71 ± 6.57) when compared to the sham groups (1 ± 0.93 and 1 ± 2.22 respectively, *p* < 0.0005 for both). TL pretreatment led to reduction in both IL-1β (3.18 ± 1.07, *p* = 0.049) and IL-6 (9.71 ± 6.57, *p* = 0.023) (Figure 6A,B).

Pro-inflammatory IFN-γ was also increased in the IRI group (5.51 ± 3.1 vs. 1.0 ± 2.02; *p* = 0.011) and TL reduced this to 1.67 ± 1.64 (*p* = 0.031 compared to vehicle) (Figure 6C). TNF-α was increased in the IRI group (2.57 ± 0.60) compared to sham (1 ± 1.05, *p* = 0.01). However, there was no significant difference between TL group (1.06 ± 0.64) and both the vehicle (*p* = 0.51) or the sham group (*p* = 0.085) (Figure 6D).

The expression of the anti-inflammatory cytokine IL-10 was higher in the TL + IRI group (14.91 ± 5.71) while levels remained low in the IRI group (4.22 ± 2.45, *p* < 0.0005) (Figure 6E). IL-13 was not different in any of the groups (Sham: 1 ± 3.38; IRI: 2.78 ± 0.93 and TL + IRI; 3.12 ± 1.98) (Figure 6F).

#### 3.2.5. TL Led to Upregulation of HO-1 in the Ileal Tissue

**Figure 7:**
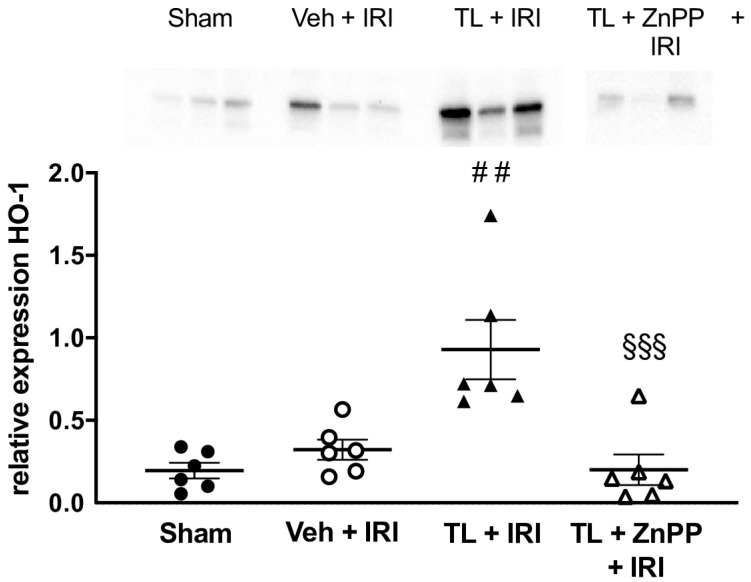
Tranilast increases HO-1 expression and ZnPP blocks it. HO-1: heme oxygenase-1; IRI: ischemia reperfusion injury; TL: tranilast; Veh: vehicle; ZnPP: zinc protoporphyrin. ##: *p* < 0.01 (compared to Vehicle); ^§§§^: *p* < 0.001 (compared to TL + IRI).

In the vehicle-treated IRI group, HO-1 levels were comparable to the results in the sham operated animals (0.34 ± 0.18 vs. 0.20 ± 0.12; *p* = 0.66). Pretreatment with TL led to an increased expression of HO-1 in the ileum (0.93 ± 0.44) compared to both the sham (*p* = 0.001) and the vehicle-treated IRI group (*p* = 0.005) (Figure 7).

### 3.3. Experiment 3: Effect of HO-1 Inhibition

#### ZnPP Abolishes the TL-Induced Upregulation of HO-1

Administration of ZnPP in addition to Tranilast (TL + ZnPP + IRI) blocked the upregulation of HO-1 to 0.20 ± 0.23 which was not different compared to IRI while at the same time being lower than the TL + IRI group (0.93 ± 0.44, *p* < 0.001) (Figure 7).

### 3.4. Inhibiting HO-1 Upregulation aBolished the Protective Effect of TL Pretreatment

**Figure 8:**
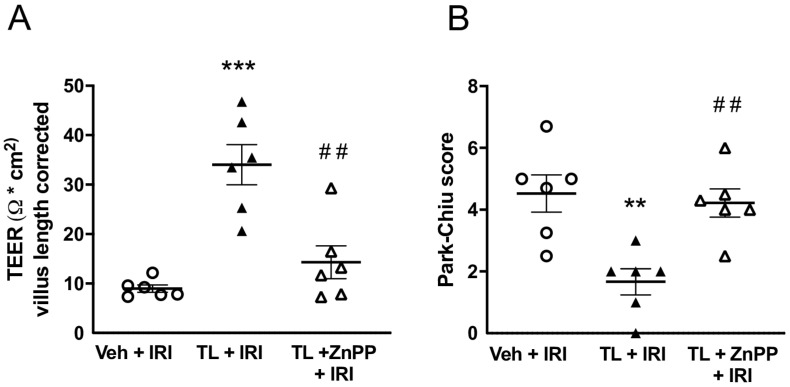
Inhibiting HO-1 upregulation abolished the protective effect of TL pretreatment as demonstrated by: (**A**) increased permeability levels measured by TEER and (**B**) increased histological damage as measured by Park-Chiu score. IRI: ischemia reperfusion injury; TEER: transepithelial electrical resistance; TL: tranilast; Veh: vehicle; ZnPP: zinc protoporphyrin. **: *p* < 0.01 (compared to vehicle); ***: *p* < 0.001 (compared to Vehicle); ^##^: *p* < 0.01 (compared to TL + IRI).

ZnPP administration limited the protective effect of TL on the intestinal barrier function by reducing the TEER to the same levels as in the IRI group (14 ± 8 Ω.cm^2^ vs. 9 ± 2 Ω.cm^2^; *p* = 0.45) (Figure 8A). Similarly, the histological damage, illustrated by the Park-Chiu score, became comparable to the vehicle-treated IRI group (5.22 ± 1.82 vs. 4.53 ± 1.48; *p* = 0.90) (Figure 8B).

## 4. Discussion

Intestinal ischemia is a devastating pathology, with an increasing prevalence due to an aging population [38]. Damage to the intestine leads to increased permeability, translocation of luminal contents and ultimately fatal sepsis [39]. For this reason, intestinal IRI is more dangerous compared to IRI in other organs. This is also the reason why ITx remains very challenging, as IRI is inevitable during the transplantation process where the resulting inflammation primes the recipient immune system, increasing the risk of rejection [19]. A recent review by Li et al. further highlighted the importance of oxidative stress in propagating ongoing injury after revascularization of ischemic intestinal tissue [40]. They showed that several important pro-inflammatory pathways are activated, such as the nuclear factor erythroid-related factor 2 (Nrf2) and the role of microRNA in either amplifying or reducing this damaging inflammation.

Our data show that TL pretreatment protects the intestine against IRI. TL consistently improves the 7-days survival from 10% to 50%. This improvement is accompanied by a reduction of both intestinal damage and the ensuing inflammation, both structurally and functionally. Moreover, our data strongly suggests that the enzyme HO-1 mediates partly the protective effect of TL.

TL was first described in 1975 as an anti-allergic drug [41]. This synthetic analog was derived from anthranilic acid, a metabolite of the indoleamine-pyrrole 2,3-dioxygenase induced tryptophan degradation [42]. TL is used in the clinic for the treatment of allergic dermatitis, rheumatoid arthritis and structuring Crohn’s disease [43,44,45]. More recently, its anti-inflammatory properties have been recognized. However, until now its potential protective effect in intestinal ischemia has not been investigated. In this study, TL reduced the levels of pro-inflammatory cytokines (especially IL-1β, IL-6 and IFN-γ) and upregulated the anti-inflammatory cytokine IL-10. We demonstrated a significant amelioration of the epithelial barrier function of the small intestine resulting in reduced endotoxin translocation and improved survival.

HO-1 upregulation seems to be the most likely protective mechanism of TL. This was demonstrated in this study, as blocking HO-1 with ZnPP prevented the protective effect of TL.

Firstly, HO-1 is a highly conserved anti-inflammatory mediator present in all cells and protects against IRI injury in several tissues, including kidney, heart and lung [31,46,47]. HO-1 is an enzyme that catalyzes the degradation of heme to biliverdin, ferrous iron, and carbon monoxide (CO). HO-1 is the primary source for endogenous CO production. Low concentrations of CO have anti-oxidant, anti-inflammatory and anti-apoptotic properties [48]. HO-1 blocks the pro-inflammatory *NF*-*κB* pathway, crucial in systemic inflammation and IRI [22]. Specifically, TL activates the mitogen-activated protein kinase (MAPK) pathways, which leads to HO-1 production. In vivo and in vitro studies have shown that HO-1, along with its major byproduct CO, inhibits pro-inflammatory cytokine expression and upregulates IL-10 [49]. In our study, we did not directly measure *NF*-*κB.* However, we could show that its downstream components—the production of pro-inflammatory cytokines—were downregulated after TL treament.

Secondly, both HO-1 and CO are powerful anti-oxidants, able to counter the effects of IRI induced ROS formation [50]. Furthermore, endotoxin and bacterial translocation leading to sepsis causes mortality after IRI. Chung et al. [51] induced fecal peritonitis in mice through a standardized cecal puncture. They showed that HO-1 deficient mice were much less resistant to the resulting peritonitis. In contrast, overexpression of HO-1 improved the ability of macrophages to clear the invading bacteria and to counteract the infection. The ability of CO, produced by the enzymatic action of HO-1, to improve phagocytosis has also been demonstrated by other groups [52,53]. This could add a ‘second line of defense’ to overcome the effects of IRI if the intestinal barrier is breached.

Finally, TL is currently primarily used as a mast cell stabilizer for anti-allergic therapy [20]. However, mast cell degranulation also plays an important role in IRI as the release of histamine, leukotrienes, and platelet-activating factors initiate part of the damaging cascade. In a rat model of intestinal IRI, mast cell deficient knock-out rats had significantly improved histology and mucosal permeability compared to wild type controls [54]. In this model, we did not measure mast cell activation which could be investigated in future experiments. Previous studies have demonstrated that HO-1 upregulation does lead to mast cell stabilization and can contribute to its anti-inflammatory effects [55]. Moreover, intestinal ischemia is mainly characterized by neutrophil and macrophage infiltration and activation in the lamina propria. Neutrophils and macrophages produce MPO, but also interleukins such as IL-1β and IL-6, TNF-α and to a lesser extent IFN-γ. The upregulation of these mediators in the present study after intestinal ischemia further supports neutrophil and macrophage activation. Since TL reduces the upregulation of IL-1β, IL-6, TNF-α, and IFN-γ (Figure 6), we can conclude that this beneficial effect is due to the reduction in the activation of neutrophils and macrophages. This finding is further supported in a preclinical model of colitis, where MPO activity was significantly reduced by TL administration [56].

The protective role of HO-1 upregulation in intestinal IRI has previously been demonstrated in two rat models [57,58]. The first used a subcutaneous hemin (an HO-1 inducing agent) injection 2 h prior to ischemia and showed improved histology and recovery of transit after 6 h. However, no difference in myeloperoxidase activity (a marker of neutrophil activation) was noted between the experimental groups. No survival or inflammatory markers were reported. In the second study, another HO-1 inducing agent (cobalt protoporphyrin) was administered intraperitoneally 24 h prior to intestinal IRI. This resulted in reduced IL-6 production, histological damage and myeloperoxidase activity. In our experiment we showed that TL-induced HO-1 upregulation also positively influenced survival, permeability, other cytokines (both pro- and anti-inflammatory) and endotoxin translocation. Furthermore, in contrast to hemin and cobalt protoporphyrin, TL has been used clinically for more than 40 years, making the use of an HO-1 therapy more feasible in the clinical practice.

Having shown that TL is effective as a pre-treatment option, the next question to address will be its efficacy as a treatment approach when given after the onset of ischemia. For this, intravenous administration of TL will be mandatory and an important hurdle is the relatively low solubility of TL in water and its poor bio-availability, requiring high dosing [59]. However, this has been addressed by a wet-milling technique leading to a new TL formulation based on nano-crystalline solid dispersion which resulted in a significant improvement in both solubility and bio-availability [60]. In future studies, this highly soluble version of TL could be used to further explore its potential as an actual treatment drug for intestinal ischemia. This aligns with the philosophy of specialized centers, which have emphasized the importance of early recognition and multimodal treatment of intestinal ischemia. In particular, Corcos et al. demonstrated substantial improvements in outcomes for patients with early pharmacological treatment (antibiotics and anticoagulation) and revascularization of intestinal ischemia [61]. In this scenario, adding an IV treatment of TL could further improve outcomes by saving marginal bowel that would otherwise have been lost.

TL could also be of particular interest in ITx, not only by reducing IRI induced damage in the transplanted bowel but also by promoting CD4+ T-regs. T cells play a crucial role in intestinal graft rejection. The activation and infiltration of cytotoxic T cells into the graft causes rejection [62]. In contrast, our group showed that expansion of T-regs (subtype: CD4^+^-CD25^+^-FoxP3) can limit intestinal graft rejection in clinical ITx [63]. The ability of TL to induce CD4+ T-regs production and reduce rejection was demonstrated in rat models of skin and cornea transplantation [64]. TL induced T-cell cycle arrest by stimulating the cell cycle-specific inhibitors p21 and p15. However, T-regs were spared from this effect and their number actually increased which may be due to differential stimulation of the aryl-hydrocarbon receptor by TL [65,66]. This immunomodulatory effect of TL has also been shown in other pre-transplant clinical models, including liver and heart [30,67] and could be of particular interest in ITx.

Despite our convincing data, a number of limitations have to be addressed [68]. Firstly, we did not investigate the pharmacokinetic of TL in our experimental animals. Instead, we relied on previous studies which demonstrated that TL is rapidly absorbed orally and achieves peak plasma concentrations after about 30 min [59]. Second, additional mechanisms beyond HO-1 upregulation may contribute to the protective effects of TL, such as mast cell stabilization, which was not investigated in this study.

## 5. Conclusions

We demonstrated that pretreatment with TL improves survival in a rat model of intestinal IRI and that this was accompanied by a substantial containment of inflammation, preservation of the intestinal barrier function, and reduced endotoxin translocation. We also showed that the protective effect of TL can be attributed to HO-1 upregulation. Future studies should investigate the efficacy of intravenously administered TL as a treatment approach for intestinal ischemia.

## Data Availability

Data are available from the authors upon reasonable request.

## Supplementary Materials

The following supporting information can be downloaded at: https://www.mdpi.com/article/10.3390/jcm14093254/s1, File S1: Sample size power calculation.

## Abbreviations

CO	carbon monoxide
HO-1	heme oxygenase-1
I-FABP	intestinal fatty acid-binding protein
IFN-γ	interferon-γ
IL	interleukin
IRI	ischemia reperfusion injury
ITx	intestinal transplantation
LPS	lipopolysaccharide
qRT-PCR	quantitative reverse-transcription polymerase chain reaction
ROS	reactive oxygen species
T-regs	regulatory T cells
TEER	trans-epithelial electrical resistance
TL	tranilast
TNF-α	tumor necrosis factor-α
WB	western blot
ZnPP	zinc protoporphyrin
