# Supplementary material for: Tranilast Reduces Intestinal Ischemia Reperfusion Injury in Rats Through the Upregulation of Heme-Oxygenase (HO)-1
Source: J Clin Med. 2025 May 7;14(9):3254. doi: 10.3390/jcm14093254 (PMC12072342; doi:10.3390/jcm14093254)
Supplement: Supplementary file 1 [file jcm-14-03254-s001.zip › jcm-3458568-supplementary.pdf]

File S1—Sample size power calculation

The rationale for the number in each group was based on power calculation which were based on predictions from the previous study from this model [28].

The primary end-point chosen was the level of histological damage, calculated by the Park-Chiu score (0-8). The expected Park-Chiu score for histological damage (range: 0-8) was 5 (±1.5) in the control group versus 2.5 in the treated group in our previous study. This resulted in a calculated group size of 6 (continuous endpoint, with an alpha of 0.05 and power of 80%).

$$k = \frac{n_2}{n_1} = 1$$

$$n_1 = \frac{(\sigma_1^2 + \sigma_2^2/K)(z_{1-\alpha/2} + z_{1-\beta})^2}{\Delta^2}$$

$$n_1 = \frac{(1.5^2 + 1.5^2/1)(1.96 + 0.84)^2}{2.5^2}$$

$$n_1 = 6$$

$$n_2 = K * n_1 = 6$$

$\Delta = |\mu_2 - \mu_1|$ = absolute difference between two means
σ_1, σ_2 = variance of mean #1 and #2
n_1 = sample size for group #1
n_2 = sample size for group #2
α = probability of type I error (usually 0.05)
β = probability of type II error (usually 0.2)
z = critical Z value for a given α or β
k = ratio of sample size for group #2 to group #1

Calculation were performed using an online sample size calculator:

https://clincalc.com/stats/samplesize.aspx

Regarding survival, the previous study showed a 0% survival in the control group versus 50% in the treatment group (FXR agonist – another protective agent against IRI). This resulted in a group size calculation of 10.

$$N_1 = \left\{ z_{1-\alpha/2} * \sqrt{\bar{p} * \bar{q} * (1 + \frac{1}{k})} + z_{1-\beta} * \sqrt{p_1 * q_1 + (\frac{p_2 * q_2}{k})} \right\}^2 / \Delta^2$$

$$q_1 = 1 - p_1$$

$$q_2 = 1 - p_2$$

$$\bar{p} = \frac{p_1 + kp_2}{1 + K}$$

$$\bar{q} = 1 - \bar{p}$$

$$N_1 = \left\{ 1.96 * \sqrt{0.25 * 0.75 * (1 + \frac{1}{1})} + 0.67 * \sqrt{0.5 * 0.5 + (\frac{0 * 1}{1})} \right\}^2 / 0.5^2$$

$$N_1 = 9$$

$$N_2 = K * N_1 = 9$$

p_1, p_2 = proportion (incidence) of groups #1 and #2
$\Delta = |p_2 - p_1|$ = absolute difference between two proportions
n_1 = sample size for group #1
n_2 = sample size for group #2
α = probability of type I error (usually 0.05)
β = probability of type II error (usually 0.2)
z = critical Z value for a given α or β
K = ratio of sample size for group #2 to group #1

Reference

28 Ceulemans, L.J.; Verbeke, L.; Decuypere, J.-P.; Farré, R.; De Hertogh, G.; Lenaerts, K.; Jochmans, I.; Monbaliu, D.; Nevens, F.; Tack, J.; et al. Farnesoid x receptor activation attenuates intestinal ischemia reperfusion injury in rats. *PLoS ONE* **2017**, *12*, e0169331. https://doi.org/10.1371/journal.pone.0169331.
